# Genetic screening of a Chinese cohort of children with hearing loss using a next-generation sequencing panel

**DOI:** 10.1186/s40246-022-00449-1

**Published:** 2023-01-04

**Authors:** Jing Ma, Xiuli Ma, Ken Lin, Rui Huang, Xianyun Bi, Cheng Ming, Li Li, Xia Li, Guo Li, Liping Zhao, Tao Yang, Yingqin Gao, Tiesong Zhang

**Affiliations:** 1 Yunnan Key Laboratory of Children’s Major Disease Research, Department of Otorhinolaryngology Head and Neck Surgery, Kunming Children’s Hospital, Kunming, China; 2 Yunnan Institute of Pediatrics, Kunming Children’s Hospital, Kunming, China; 3 Department of Otolaryngology-Head and Neck Surgery, Shanghai Ninth People’s Hospital, Shanghai Jiao Tong University School of Medicine, Shanghai, China

**Keywords:** Hearing loss, Molecular diagnostics, Next-generation sequencing, Gene panels

## Abstract

**Background:**

At present, the hereditary hearing loss homepage, (https://hereditaryhearingloss.org/), includes 258 deafness genes and more than 500 genes that have been reported to cause deafness. With few exceptions, the region-specific distributions are unclear for many of the identifie*d variants and genes.*

**Methods:**

Here, we used a custom capture panel to perform targeted sequencing of 518 genes in a cohort of 879 deaf Chinese probands who lived in Yunnan. Mutation sites of the parents were performed by high-throughput sequencing and validated by Sanger sequencing.

**Results:**

The ratio of male to female patients was close to 1:1 (441:438) and the age of onset was mainly under six. Most patients (93.5%) were diagnosed with moderate to severe deafness. Four hundred and twenty-eight patients had variants in a deafness gene, with a detection rate of 48.7%. Pathogenic variants were detected in 98 genes and a number of these were recurrent within the cohort. However, many of the variants were rarely observed in the cohort. In accordance with the American College of Medical Genetics and Genomics, pathogenic, likely pathogenic and variants of uncertain significance accounted for 34.3%, 19.3% and 46.4% of all detected variants, respectively. The most common genes included *GJB2*, *SLC26A4*, *MYO15A*, *MYO7A*, *TMC1*, *CDH23*, *USH2A* and *WFS1*, which contained variants in more than ten cases. The two genes with the highest mutation frequency were *GJB2* and *SLC26A4*, which accounted for 28.5% (122/428) of positive patients. We showed that more than 60.3% of coding variants were rare and novel. Of the variants that we detected, 80.0% were in coding regions, 17.9% were in introns and 2.1% were copy number variants.

**Conclusion:**

The common mutation genes and loci detected in this study were different from those detected in other regions or ethnic groups, which suggested that genetic screening or testing programs for deafness should be formulated in accordance with the genetic characteristics of the region.

**Supplementary Information:**

The online version contains supplementary material available at 10.1186/s40246-022-00449-1.

## Background

Hereditary hearing loss (HL) is one of the most common sensory disorders, with an incidence of 1–2 cases per 1000 newborns [1]. In 2021, the World Health Organization reported that more than 1.5 billion people experience some decline in their hearing capacity during their life course and at least 430 million will require care [2]. Hereditary hearing loss is nonsyndromic in 70% of newborns. The remaining 30% present with the syndromic form and exhibit additional abnormalities [3]. Deafness is a disease with high genetic and clinical heterogeneity. Clinical diagnosis can determine the degree of deafness but cannot clarify the cause of the disease. The molecular etiology of most patients can be identified by a combination of genetic testing and clinical diagnosis [3, 4]. The introduction of next-generation sequencing (NGS) has resulted in great progress in the diagnostics of HL [5, 6]. As of July 2022, mutations in 258 different genes have been reported to cause HL (http://hereditaryhearingloss.org). Genetic factors account for more than 50% of cases, in which the majority exhibit autosomal recessive inheritance (75–80%). Twenty to twenty-five percent show autosomal dominant inheritance and 1.0-1.5% show X-linked or mitochondrial inheritance [7].

To date, the genes involved and the spectrum of mutations are largely unknown in early HL patients from Yunnan. In this study, we presented the results of an NGS analysis of a large cohort of 879 patients with deafness. Using this large number of patients, we expected to reveal the genes and variants that cause HL in the Yunnan region.

## Materials and methods

### Subjects

This study was approved by the Ethics Committee of Kunming Children's Hospital (Kunming, China). Informed consent was obtained from the parents of the patients, prior to sampling. The diagnosis of HL was established via standard audiometry, in a soundproofed room, in accordance with the current clinical standards. Hearing loss was classed as either congenital onset or prelingual onset and the severity was established as mild (between 25 and 40 dB), moderate (between 40 and 70 dB) or severe/profound (> 70 dB). Clinical evaluation included a thorough physical examination and otoscopy, in all cases. Additional evaluations, which included a high-resolution, thin-section computed tomography (CT) and magnetic resonance imaging (MRI) of the temporal bone, were performed when possible. We extracted DNA from peripheral blood leukocytes of the probands, in accordance with standard procedures.

### DNA extraction, library construct, sequencing and quality control

EDTA anti-coagulated venous blood (3 ml) were collected from a cohort of 879 deaf Chinese probands and the parents of 357 patients. The genomic DNA was extracted using the QIAamp DNA Blood Mini Kit following the manufacturer’s protocol. Then make the DNA fragmentaion after quality control. DNA sequencing library was constructed by NEXTflex Rapid DNA-Seq Kit and Deaf-gene Panel probes were used for library capture. Samples were analyzed using a 518 gene panel (Additional file 1: Table S1), on the Illumina Next Generation Sequencing platform. The 518 gene panel was a probe capture panel that was comprised of non-syndromic deafness genes (such as *GJB2* and *GJB3*), syndromic deafness genes (such as *SLC26A4*, *FOXI1* and *EDN3*) and mitochondrial deafness genes (such as *MT-TS1*, *MT-RNR1*, *MT-TL1*, *MT-TK* and *MT-TE*).The prepared library was loaded onto NovaSeq 6000 (Illumina, CA, United States). The average coverage was ≥ 100, the percentage of target region ≥ 20 × was ≥ 98%, and, and Q20 ≥ 95% were set as the sequencing quality parameters.

### Bioinformation analysis

The sequenced reads were aligned to the reference genome (hg19) using BWA MEN, and PCR duplicates were marked with PICARD. Variants were called by HaplotypeCaller in GATK4.0 with default parameters, and retained considering DP (reads depth) ≥ 10, MQ (Mapping Quality) ≥ 30 and GQ(Genotyping Quality) ≥ 20. After annotation using ANNOVAR, variants both in coding region and splicing site were kept and synonymous variants were removed.

### Variant curation

Deafness-Associated Genes Panel can obtain about 5 thousand variants in one patient and most of the variants are benign. Those variants can be screened through database of normal crowd (gnomAD, threshold is set as 0.1%) and Computational (in silico) predictive (Revel). About 100 are selected. And then screened by variant types, genetic mode, HGMD and ClinVar database to confirm several target variants. And the target variants are curationed according to the American College of Medical Genetics and Genomics (ACMG) [8] and the Association for Molecular Pathology (AMP) guidelines, the ClinGen SVI general recommendations and the ClinGen VCEP recommendations. Using the following evidence: (1) the frequency in the normal population (gnomAD database); (2) computational prediction: mutations were predicted using Pubvar (https://www.pubvar.com/), with several prediction software packages, such as Polyphen, SIFT and MutationTaster; (3) literature reports: target variants were searched in HGMD, ClinVar and Pubmed. The variants are classified into Pathogenic (P), Likely Pathogenic (LP), Benign (B), Likely Benign (LB), and Uncertain significance (VUS).

### Sanger sequencing

The target variants were validated by Sanger sequencing. Sequencing primers were designed using online Primer Designer Tool and compared with those from public databases to avoid SNPs and nonspecific amplification. Genomic DNA was amplified using the HotStarTaq Master Mix Kit with the following conditions: 95 °C for 15 min; followed by a program of 94 °C for 30 s, 60 °C for 30 s, and 72 °C for 45 s for 35 cycles; and ending with a 10-min extension at 72 °C. Amplication program was different according to different target DNA sequence. Amplicons were purified using ExoSAP-IT Express and bidirectionally sequenced using BigDye Terminator version 1.1 on an ABI3730xl DNA analyzer. Data analysis was conducted using Applied Biosystems Minor Variant Finder.

## Results

### Patients

We sequentially accrued 879 HL patients during the study period. There were no exclusion criteria. As shown in Table 1, the demographics of the patients were binned into five categories: Sex, onset, severity, family history and physical exam. There were 72 patients who did not provide clinical information. The ratio of male to female patients was close to 1:1 (441:438) and the largest group for the age of onset data was ‘under the age of six’. The most common characteristics included: young age (86.2% were < 18 years of age) and severe-to-profound HL (44.5%). Most patients had a normal physical exam (81.8%) and approximately one-third had no family history of HL (37.3%).

**Table 1:** Phenotypic characteristics of patients evaluated in this study

Characteristic	Number	%
*Sex*		
Male	441	50.2
Female	438	49.8
*Age when onset (years)*		
Age < 6	406	46.2
Age 6–18	387	44.0
Age > 18	86	9.8
*Family history*		
Autosomal recessive	260	29.6
Autosomal dominant	160	18.2
X-linked	10	1.1
Ambiguous	5	0.6
No family history	329	37.4
Not provided	113	12.9
*Severity*		
Mild-moderate	57	6.5
Severe-profound	822	93.5
*Physical exam*		
Normal	719	81.8
Any abnormality	73	8.3
Not provided	87	9.9

### Molecular findings among probands

**Fig. 1:**
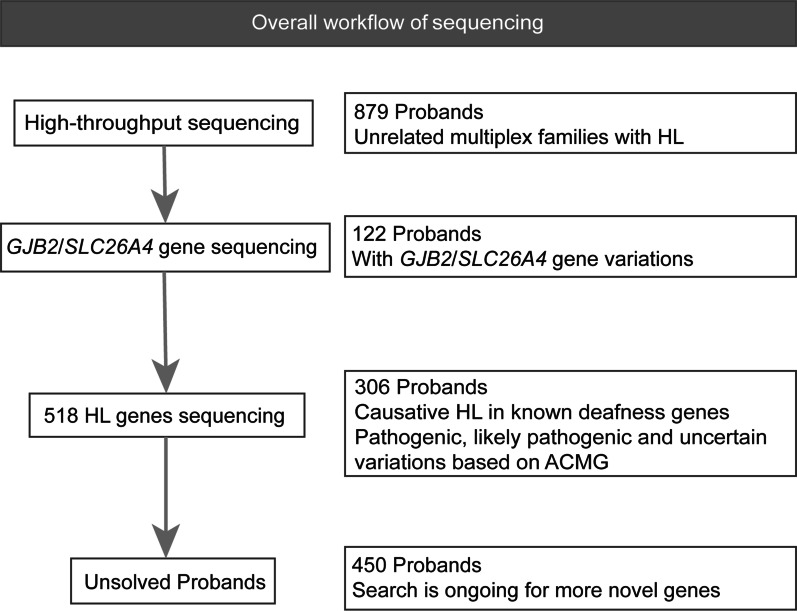
Overall workflow of sequencing. *HL* hearing loss, *WES* whole exome sequencing, *ACMG* American College of Medical Genetics and Genomics

**Fig. 2:**
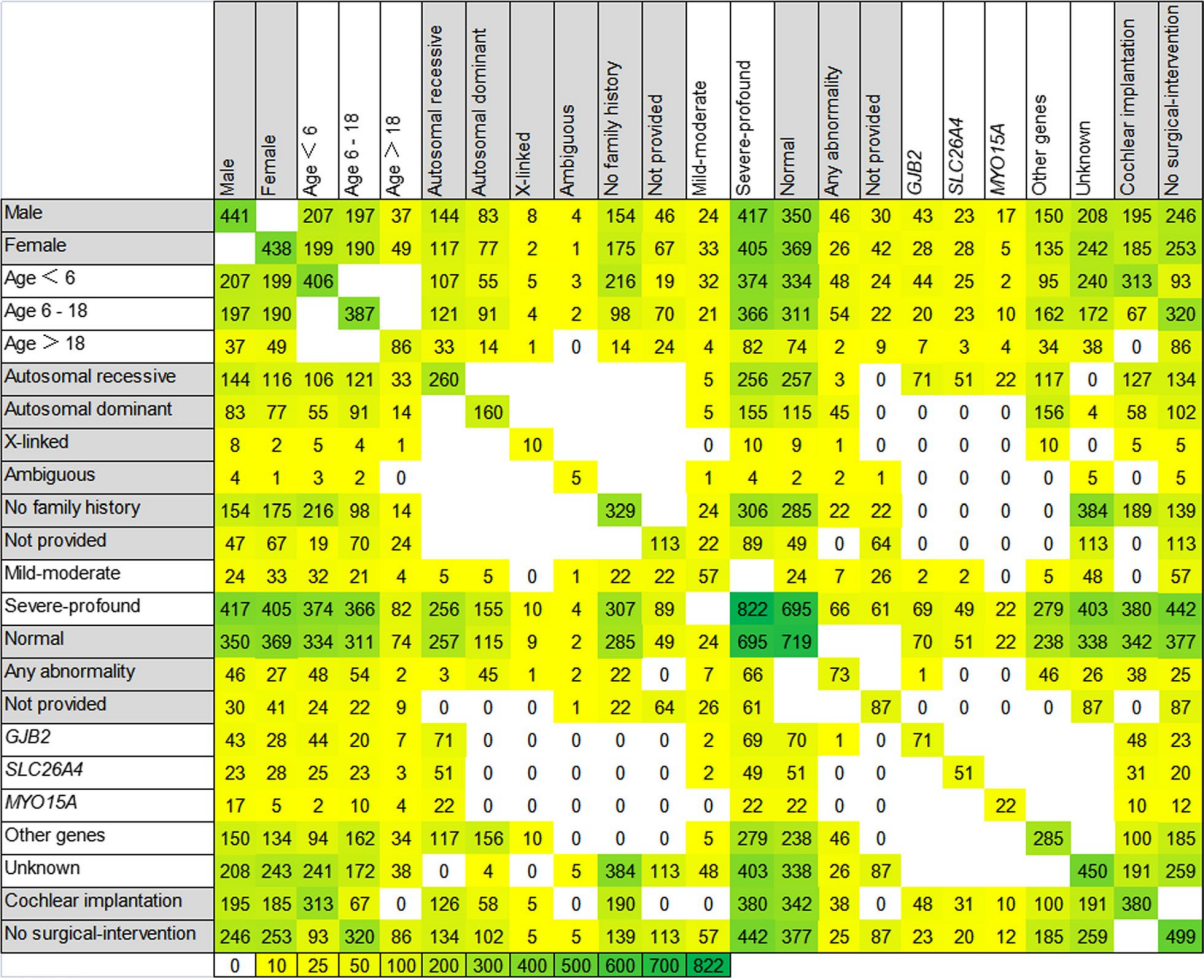
The interaction graph of basic and diagnostic information for each sample. Positive diagnosis is influenced by ethnic, clinical, and phenotypic characteristics in sporadic hearing loss population. *N* for each combination of two reported characteristics for all combinations. Color/shading reflects the number of patients with the paired criteria, up to the maximum of *n* = 822. *AD* autosomal dominant, *AR* autosomal recessive

As shown in Fig. 1, 428 out of the 879 (48.7%) patients had mutations (Additional file 2: Table S2) in known HL genes, which were classified as pathogenic (P), likely pathogenic (LP) or variants of uncertain significance (VUS), in accordance with ACMG. The basic and diagnostic information for each sample were shown in Fig. 2. The *GJB2* and *SLC26A4* genes were the most common deafness susceptibility genes and their mutations accounted for 28.5% [*GJB2:* 16.6% (71/428), *SLC26A4:* 11.9% (51/428)] of mutation positive patients in our study. Three hundred and six patients (71.5%) had others known deafness gene variants. The molecular etiology of the remaining 450 patients was not identified. In our analysis of the detected genetic variants, we have used a healthy control group from the Chinese population that included WES data from 5000 normal people provided by MyGenostics Inc (Inhouse). All the mutation sites in this study have relatively low carry rates in the normal population (except *GJB2* and *SLC26A4*, Additional file 3: Table S3), and approximately 36.4% of the VUS variants in this study had zero carry rates in healthy controls (Additional file 3: Table S3).

Using the pedigree analysis and NGS results, we observed autosomal dominant inheritance for 160 of the 428 deafness gene variants (160/428, 37.4%) and autosomal recessive inheritance for 260 variants (260/428, 60.7%). We observed X-linked inheritance in 10 cases (10/428, 2.3%) and mitochondrial gene mutation in two cases (2/428, 0.5%). In this cohort, sequence variants were identified in a total of 98 genes (Table 2). The genes that were identified in at least five patients included *GJB2* (16.6%; 71/428), *SLC26A4* (11.9%; 51/428), *MYO15A* (5.1%; 22/428), *MYO7A* (4.9%; 21/428), *TMC1* (3.7%; 16/428), *CDH23* (3.3%; 14/428), *MITF* (3.0%; 13/428), *USH2A* (3.0%; 13/428), *WFS1* (2.6%; 11/428), *COL11A2* (2.3%; 10/428), *OTOF* (2.3%; 10/428), *SOX10* (2.3%; 10/428), *PTPN11* (1.9%; 8/428), *OTOG* (1.4%; 6/428), *TNC* (1.4% 6/428), *EYA1* (1.2%; 5/428), *GJB3* (1.2%; 5/428) and *TECTA* (1.2%; 5/428).

**Table 2:** The deafness gene detected in this cohort

No	Gene	Number of case		No	Gene	Number of case		No	Gene	Number of case
1	*GJB2*	71		34	*TRRAP*	3		67	*GAB1*	1
2	*SLC26A4*	51		35	*ADGRV1*	3		68	*CREBBP*	1
3	*MYO15A*	22		36	*FGFR3*	2		69	*FOXC1*	1
4	*MYO7A*	21		37	*MT-RNR1*	2		70	*CLDN14*	1
5	*TMC1*	13		38	*GATA3*	2		71	*COL1A1*	1
6	*CDH23*	14		39	*SALL4*	2		72	*COL9A1*	1
7	*MITF*	13		40	*POU3F4*	2		73	*TRPV4*	1
8	*USH2A*	13		41	*CHD7*	2		74	*FLNB*	1
9	*WFS1*	11		42	*TSHZ1*	2		75	*COL2A1*	1
10	*COL11A2*	10		43	*MYO6*	2		76	*HOMER2*	1
11	*OTOF*	10		44	*COL4A6*	2		77	*LARS2*	1
12	*SOX10*	10		45	*USH1G*	2		78	*DSPP*	1
13	*PTPN11*	8		46	*DIAPH3*	2		79	*CLRN1*	1
14	*OTOG*	6		47	*EYA4*	2		80	*SH3TC2*	1
15	*TNC*	6		48	*DIAPH1*	2		81	*MARVELD2*	1
16	*EYA1*	5		49	*EDNRB*	1		82	*P2RX2*	1
17	*GJB3*	5		50	*PRPS1*	1		83	*SIX5*	1
18	*TECTA*	5		51	*ESRRB*	1		84	*CEACAM16*	1
19	*BDP1*	4		52	*ACTG1*	1		85	*TBL1Y*	1
20	*USH1C*	4		53	*GRXCR1*	1		86	*YAP1*	1
21	*PTPRQ*	4		54	*ACTB*	1		87	*CABP2*	1
22	*TRIOBP*	4		55	*TWIST1*	1		88	*CD164*	1
23	*MYO1A*	4		56	*PDE1C*	1		89	*GSDME*	1
24	*PCDH15*	4		57	*TMIE*	1		90	*MYH14*	1
25	*WHRN*	4		58	*ANKRD11*	1		91	*TCOF1*	1
26	*DMXL2*	3		59	*AIFM1*	1		92	*POLG*	1
27	*MYH9*	3		60	*TPRN*	1		93	*NLRP3*	1
28	*LHFPL5*	3		61	*PDZD7*	1		94	*REST*	1
29	*MCM2*	3		62	*FLNA*	1		95	*PAX3*	1
30	*TBC1D24*	3		63	*BCOR*	1		96	*KCNQ4*	1
31	*COL11A1*	3		64	*TNFRSF11A*	1		97	*COL4A5*	1
32	*LOXHD1*	3		65	*LRP2*	1		98	*CCDC50*	1
33	*SLC17A8*	3		66	*ERCC6*	1				

**Fig. 3:**
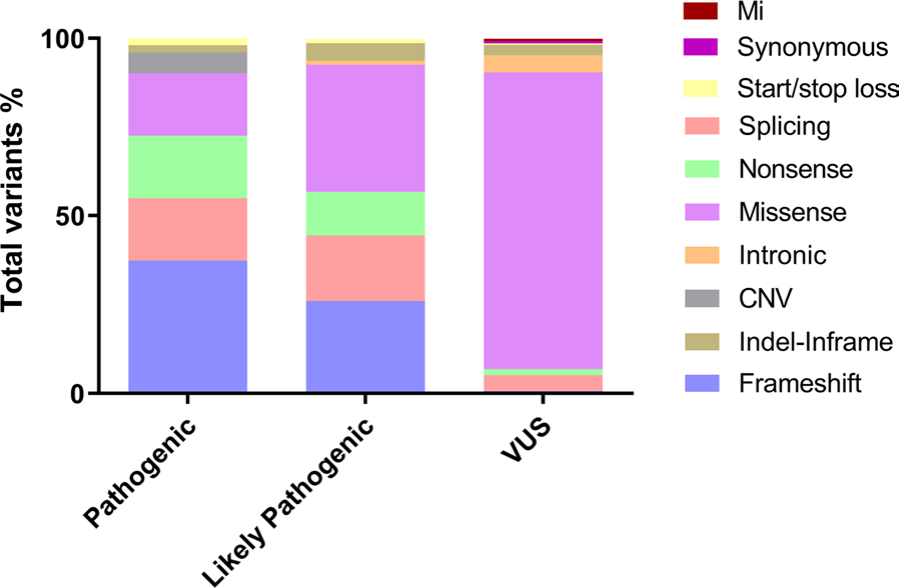
Detected deafness variant genes are rated with reference to ACMG. *VUS* variant of uncertain significance, *Mi* mitochondrion, *CNV* copy-number variations

Analysis of NGS data indicated that 428 patients had variants that could be classified as P, LP or VUS, based on ACMG guidelines. These accounted for 34.3%, 19.3% and 46.4% of variants, respectively. Of the 428 variants, 42.3% had previously been reported in the literature, whilst 57.7% were novel. Variants that led to protein truncation, such as frameshift mutations and nonsense mutations, were found at a higher rate and were usually classed as P or LP (Fig. 3). In addition, missense mutations or splice mutations were also detect in large numbers and rated as P or LP. It was noted that a small number of copy number variations (CNVs) were detected among the pathogenic variants. However, the proportion of mutation types in the VUS class was quite different from that of the P and LP classes. Missense mutations were the most common mutation type in the VUS class and most had not been included in the database or previously reported in the literature.

**Fig. 4:**
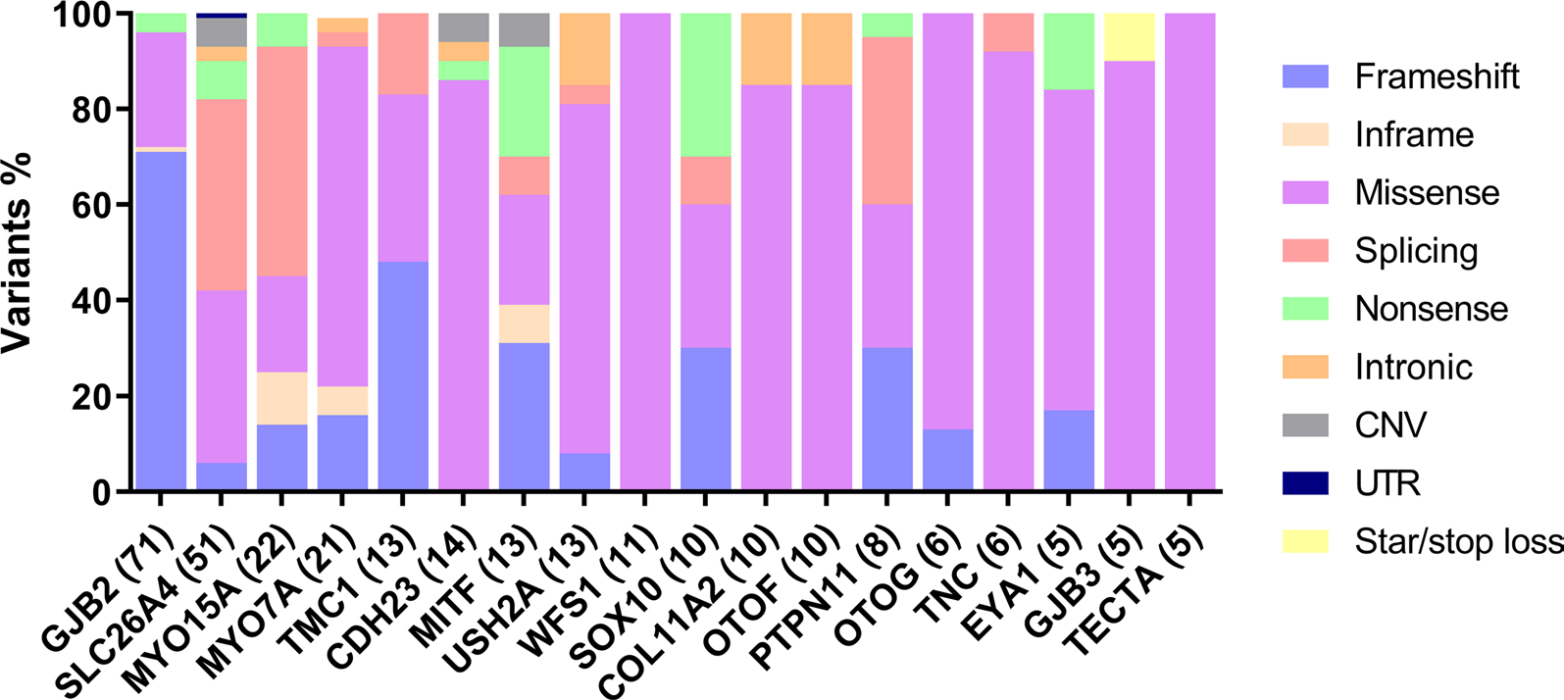
Mutation types of gene whose detected was or more than 5 probands in this deafness cohort. *UTR* untranslated region, *CNV* copy-number variations

Using the mutation types found in more than five probands in this deafness cohort, it was observed that the proportion of missense mutations was the highest, followed by frameshift mutations and splicing mutations (Fig. 4). We also noted a wide variation across genes in the fractional contribution of missense versus loss of function (LOF) variants (frameshift and nonsense) (Fig. 4). Some genes had exclusively missense mutations (*WFS1* and *TECTA*, Fig. 4).

The proportion of CNVs was 2.1% (Fig. 4). Six compound heterozygous CNVs were identified in *SLC26A4* and two heterozygous CNVs were identified in *EYA1* and *MITF*. One novel, homozygous CNV, which was a deletion that spanned exons 37–50 of the *CDH23* gene, was identified in this cohort. Deleted exons did not amplify in confirmatory PCR reactions from proband samples.

## Discussion

The development of the Human Genome Project has promoted the combination of genetics and clinical medicine for diagnostics. The emergence of genetic screening and diagnosis for deafness is of great significance for the clarification of the cause of deafness and to prevent its occurrence [9–11]. Since the 1980s, with the development of technology, great progress has been made in the study of the genetic etiology of deafness. To date, more than 258 deafness genes have been cloned or identified (https://hereditaryhearingloss.org/) and it has been reported that more than 500 genes may contribute to the deafness phenotype. However, the diagnosis of hereditary deafness is extremely challenging for the following reasons: Firstly, hereditary deafness is a disease with very high genetic, clinical and ethnic heterogeneity. Secondly, hereditary deafness genes may have different categories of genetic variants, such as single nucleotide variations (SNVs), insertions and deletions (INDELs) and CNVs. Thirdly, most deafness gene variants do not occur repeatedly.

The introduction of NGS into the field of medical genetics has opened up new horizons for clinical diagnosis and scientific research and it has become a powerful auxiliary technology. Over the past decade, studies have attempted to enrich the known HL genes using capture-based or PCR-based targeted enrichment methods, followed by NGS, to screen for variants [12]. It can be concluded that the sensitivity and specificity of NGS for HL genetic detection is comparable to conventional Sanger sequencing, based on previous studies. Recently, Gema García-García et al. [13] detected the variants responsible for the disease in 47 out of 118 families (40%), with a custom panel that included 59 genes associated with non-syndromic or syndromic HL. Seco et al. [14] published a study that evaluated the diagnostic utility of NGS targeting using a gene panel of 120 HL-related genes. Using this strategy, they were able to identify a causative variant of HL in 67 out of 200 patients (33.5%). In this study, 518 deafness-related genes were identified in 879 patients. Deafness gene variants were detected in 428 children and the detection rate was 48.7%, which was slightly higher than the detection rates observed when using the smaller gene panels that contain the common deafness-related variants. Our study showed that the more deafness-related genes that are included, the higher the detection rate, which indicates that some populations have rare variants. These results are significant for the molecular diagnosis of deafness and further research.

With the continuous development of gene sequencing technology, the ability of variation detection far exceeds the ability of clinical interpretation, more and more VUS variants have been detected and reported [15–17]. In this study, 46.4% of those detected mutation sites were rated as VUS, which accounted for almost half of the total mutations. However, the majority of gene variants assessed as VUS are very rare, with low detection rate in human, making it difficult to interpret such variants. In this study, about 90% of the VUS variants are missense mutations, which may often be considered to causes a slight impact because they only cause one amino acid change. In fact, missense mutations may be the main types of mutations in some genes. According to the HGMD database, about 89.0% of the variants in *CDH23* gene, 85.0% of the variants in *TECTA* gene, and 69.4% of the variants in *OTOF* gene are missense mutations, which reminds us that the missense mutations in deafness causative genes should be given more consideration. With the deepening of research and the accumulation of deafness sequencing data, some databases have been established, such as the Deafness Variation Database (DVD) [17], which can re-adjust the ACMG ranking of variants. The clinical diagnostic value of VUS may be considered by intergrating the ranking results of different databases. In addition, McDiarmid [18] used CRISPR-Cas9 genome engineering technique to modify model organisms to study the association of VUS with disease. Therefore, some variants are classified as VUS may only be temporary for now. With the decrease of sequencing cost, more and more sequencing results of deaf people or even normal people will be studied. The accumulation of sequencing data will help more accurate analysis and ranking of variations, which are why we include VUS variables into this study. What needs to be emphasized here is that although we report these VUS mutations, the pathogenicity is still unclear. The clinical reference to these variants in the diagnosis of deafness requires great caution.

Deafness is a disease with high genetic heterogeneity and one of the manifestations is that the high-frequency mutation genes are different in different populations. The *GJB2* and *MYO6* genes are the most commonly mutated HL genes in the global population [1, 19]. In Czech patients, the common HL genes are *GJB2* and *STRC* [20, 21]. However, in the Chinese population, the common HL genes are *GJB2* and *SLC26A4* [9]. These studies show that molecular diagnostic analyses are highly population dependent, as the mutation spectrum for HL can differ greatly between populations. In this study, variants were detected in ten genes (*GJB2*, *SLC26A4*, *MYO15A*, *MYO7A*, *TMC1*, *CDH23*, *MITF*, *USH2A*, *WFS1* and *SOX10*), which explained 55.7% of patients with deafness variants. With the exception of mutations in two relatively common genes, *GJB2* (16.6%) and *SLC26A4* (11.9%), most reported mutations were present in only a single or a small number of families. After excluding *GJB2* and *SLC26A4* mutations, we detected P, LP or VUS mutations in the known HL genes in 34.9% of the probands.

Our results demonstrated that NGS screening is a powerful technology for the identification of mutations in HL genes. From an epidemiological perspective, *GJB2* and *SLC26A4* mutations were involved in 28.5% of cases of deafness with a gene variant, while the remaining cases arose from rare gene mutations. For instance, *CDH23*, *PCDH15* and *MYO7A* are large genes and it appears that these genes are more prone to mutations [22, 23]. The mutations in some genes did not appear repeatedly. For example, none of the mutations in the *SOX10* and *MITF* genes were recurrent in our cohort. This may indicate that *SOX10* and *MITF* are relatively more prone to de novo mutations or that they are highly conserved genes in which variation is rarely tolerated. Some genes, such as *TNIE* and *TPRN,* had less than 20 loci reported in the HGMD database. In these two genes, only one case of a rare gene mutation was detected in our cohort. In contrast, four patients in our study had *BDP1* gene mutations, despite this gene having less than 20 mutation sites in the HGMG database. It is worth noting that, among these four individuals, three had the same mutation, which has not been reported in the literature. This suggested that this locus may be a funder mutation, as they are all from Zhaotong city of Yunnan Province in southwest China.

Mutations in introns may be relatively common in deafness patients. Among the 98 deafness-related genes detected in this study, *SLC26A4* (62.7%, 32/51), *MYO15A* (63.6%, 14/22), *OTOF* (40.0%, 4/10) and *PTPRQ* (75.0%, 3/4) contained intronic variants. In previous studies, it has been confirmed that the intron of *SLC26A4* is prone to mutations, especially at the c.919-2A > G site, which is one of the mutation hotspots in the Asian population [24, 25]. Our research also confirmed this. The variants detected in this study included common intronic mutations and also some rare or novel variants. This suggested that there are some unknown variant sites that require comprehensive testing, in order to find more genetic variants.

Copy number variants are a major contributor to hereditary HL, which shows the importance of the inclusion of CNV detection in a molecular diagnostic analysis for HL [26, 27]. Although there have been studies that show the importance of CNV analysis of NGS data, in the etiology of deafness, this analysis method is still being optimized for clinical use. The study by Shearer et al. [28] used targeted enrichment and NGS with integrated CNV detection for HL in 686 patients. At least one CNV within a known deafness gene was observed in 15.2% (104) of patients. In our cohort, we used NGS to identify CNVs in four different genes (*SLC26A4*, *MITF*, *EYA1* and *CDH23*), in nine patients (2.1%, 9/428). Several studies have shown that *STRC* and *OTOA* are two of the deafness genes that are more prone to CNVs [28–30]. However, we only detected a heterozygous deletion in exons 2–9 of the *STRC* gene in one patient. Because the variant is not autosomal recessive, this patient is a carrier. Therefore, it was not included in the positive results of this study. We did not detect CNV in the *OTOA* gene, which may be caused by regional differences. These results demonstrated that NGS can detect CNVs but more medical records and studies are needed to confirm whether all CNVs can be detected [31].

This study was not able to fully identify and explain the molecular etiology of all patients. Deafness-related gene variants were not detected in 20.8% of the 879 patients. These patients were likely to have undetected variants but more research is needed to confirm this speculation. It was noted that 43.2% of the unsolved patients had a pathogenic variant in a known HL gene. These patients may have had another mutation that had not been detected, or they may have been carriers who had developed deafness [32, 33]. Deafness can also occur through an oligogenic pattern of inheritance, were two or more pathogenic variants in different genes are detected in individuals with unsolved HL. Combinations of some variants have been reported to be the cause of oligogenesis, such as variants in *SLC26A4* and *KCNJ10*, or *SLC26A4* and *FOXI1* [34]. However, we did not find any patients with the previously reported combinations of variants.

In this study, we applied NGS technology to (1) identify the molecular epidemiological etiology of HL in Chinese children and (2) discover novel mutations in causative genes. The results of the study provide a reference for the development of genetic screening or diagnosis of deafness, which is suitable for this region. In our analysis, we only considered coding regions, which is a limitation of this study. Pathogenic variants in noncoding regions or transcripts may also cause HL. In addition, due to the lack of family history, it was not clear whether the deafness gene variants carried by some patients contributed to the development of the disease.

Our data indicated that many rare variants are responsible for HL in this cohort and these mutations can be detected using NGS, which is approximately comparable in accuracy to Sanger sequencing. It is likely that there are undetected, rare variants that are specific to the Chinese population. Therefore, the main challenge for the future will be the establishment of population specific mutation-spectra, to achieve accurate, personalized, comprehensive molecular testing for HL.

## Supplementary Information

**Additional file 1: Table S1.** The panel containing 518 genes associated with deafness.

**Additional file 2: Table S2.** Mutations identified in known hearing loss genes.

**Additional file 3: Table S3.** The frequency of mutation site in 5000 normal population.

## Data Availability

The datasets used and/or analyzed during the current study are available from the corresponding author on reasonable request.

## Abbreviations

HL: Hereditary hearing loss
NGS: Next-generation sequencing
MRI: Magnetic resonance imaging
ACMG: American College of Medical Genetics and Genomics
P: Pathogenic
LP: Likely pathogenic
VUS: Variants of uncertain significance
CNVs: Copy number variations
LOF: Loss of function
SNVs: Nucleotide variations
INDELs: Insertions and deletions
