# Supplementary material for: Genetic screening of a Chinese cohort of children with hearing loss using a next-generation sequencing panel
Source: Hum Genomics. 2023 Jan 4;17:1. doi: 10.1186/s40246-022-00449-1 (PMC9811745; doi:10.1186/s40246-022-00449-1)
Supplement: Supplementary file 2 — Additional file 2: Table S2. Mutations identified in known hearing loss genes. [file 40246_2022_449_MOESM2_ESM.docx]

**Table S2** Mutations identified in known hearing loss genes

|  | **Gene** | **Loci** | **cDNA** | **Protein** | **Zygosity Hom/Het** | **Variation type** | **ACMG** | **Inheritance** |
| --- | --- | --- | --- | --- | --- | --- | --- | --- |
| 8 | *GJB2* | Exon2 | c.235delC | p.L79Cfs*3 | Hom | Frameshift | P | AR |
| 64 | *GJB2* | Exon2；Exon2 | c.167delT； c.176_191del | p.L56Rfs*26；p.G59Afs*18 | Het；Het | Frameshift；Frameshift | P；P | AR |
| 149 | *GJB2* | Exon2；Exon2 | c.299_300delAT；c.235delC | p.H100Rfs*14；p.L79Cfs*3 | Het；Het | Frameshift；Frameshift | P；P | AR |
| 156 | *GJB2* | Exon2 | c.235delC | p.L79Cfs*3 | Hom | Frameshift | P | AR |
| 161 | *GJB2* | Exon2；Exon2 | c.176_191del；c.235delC | p.G59Afs*18；p.L79Cfs*3 | Het；Het | Frameshift；Frameshift | P；P | AR |
| 199 | *GJB2* | Exon2；Exon2 | c.605_606insAGAAGACTGTCTTCACAGTGTTCATGATTGCAGTGTCTGGAATTTG；c.299_300del | p.C202_I203delinsX；p.H100Rfs*14 | Het；Het | Nonsense；Frameshift | P；P | AR |
| 203 | *GJB2* | Exon2；Exon2 | c.235delC；c.598G>A | p.L79Cfs*3；p.G200R | Het；Het | Frameshift；Missense | P；P | AR |
| 254 | *GJB2* | Exon2 | c.235delC | p.L79Cfs*3 | Hom | Frameshift | P | AR |
| 257 | *GJB2* | Exon2 | c.235delC | p.L79Cfs*3 | Hom | Frameshift | P | AR |
| 262 | *GJB2* | Exon2 | c.235delC | p.L79Cfs*3 | Hom | Frameshift | P | AR |
| 318 | *GJB2* | Exon2；Exon2 | c.235delC；c.512_513insAACG | p.L79Cfs*3；p.A171Efs*40 | Het；Het | Frameshift；Frameshift | P；P | AR |
| 351 | *GJB2* | Exon2；Exon2 | c.235delC；c.109G>A | p.L79fs*3；p.V37I | Het；Het | Frameshift；Missense | P；P | AR |
| 396 | *GJB2* | Exon2；Exon2 | c.508_511dupAACG；c.235delC | p.A171fs*40；p.L79fs*3 | Het；Het | Frameshift；Frameshift | P；P | AR |
| 404 | *GJB2* | Exon2；Exon2 | c.235delC；c.299_300delAT | p.L79Cfs*3；p.H100Rfs*14 | Het；Het | Frameshift；Frameshift | P；P | AR |
| 408 | *GJB2* | Exon2 | c.235delC | p.L79Cfs*3 | Hom | Frameshift | P | AR |
| 414 | *GJB2* | Exon2；Exon2 | c.299_300delAT；c.235delC | p.H100Rfs*14；p.L79Cfs*3 | Het；Het | Frameshift；Frameshift | P；P | AR |
| 430 | *GJB2* | Exon2 | c.235delC | p.L79Cfs*3 | Hom | Frameshift | P | AR |
| 432 | *GJB2* | Exon2；Exon2 | c.508_511dupAACG；c.235delC | p.A171Efs*40；p.L79Cfs*3 | Het；Het | Frameshift；Frameshift | P；P | AR |
| 435 | *GJB2* | Exon2；Exon2 | c.235delC；c.478G>A | p.L79Cfs*3；p.G160S | Hom；Hom | Frameshift；Missense | P；P | AR |
| 439 | *GJB2* | Exon2；Exon2 | c.508_511dupAACG；c.235delC | p.A171Efs*40；p.L79Cfs*3 | Het；Het | Frameshift；Frameshift | P；P | AR |
| 440 | *GJB2* | Exon2 | c.235delC | p.L79Cfs*3 | Hom | Frameshift | P | AR |
| 441 | *GJB2* | Exon2；Exon2 | c.9G>A；c.235delC | p.W3X；p.L79Cfs*3 | Het；Het | Nonsense；Frameshift | P；P | AR |
| 448 | *GJB2* | Exon2 | c.235delC | p.L79Cfs*3 | Hom | Frameshift | P | AR |
| 493 | *GJB2* | Exon2；Exon2 | c.299_300delAT；c.235delC | p.H100Rfs*14；p.L79Cfs*3 | Het；Het | Frameshift；Frameshift | P；P | AR |
| 502 | *GJB2* | Exon2 | c.235delC | p.L79Cfs*3 | Hom | Frameshift | P | AR |
| 813 | *GJB2* | Exon2 | c.235delC | p.L79Cfs*3 | Hom | Frameshift | P | AR |
| 865 | *GJB2* | Exon2 | c.235delC | p.L79Cfs*3 | Hom | Frameshift | P | AR |
| 832 | *GJB2* | Exon2 | c.235delC | p.L79Cfs*3 | Hom | Frameshift | P | AR |
| 840 | *GJB2* | Exon2；Exon2 | c.299_300delAT；c.109G>A | p.H100Rfs*14；p.V37I | Het；Het | Frameshift；Missense | P；P | AR |
| 366 | *GJB2* | Exon2 | c.235delC | p.L79Cfs*3 | Hom | Frameshift | P | AR |
| 358 | *GJB2* | Exon2；Exon2 | c.511_512insAACG；c.235delC | p.A171Efs*40；p.L79Cfs*3 | Het；Het | Frameshift；Frameshift | P；P | AR |
| 350 | *GJB2* | Exon2 | c.235delC | p.L79Cfs*3 | Hom | Frameshift | P | AR |
| 339 | *GJB2* | Exon2；Exon2 | c.511_512insAACG；c.235delC | p.A171Efs*40；p.L79Cfs*3 | Het；Het | Frameshift；Frameshift | P；P | AR |
| 336 | *GJB2* | Exon2 | c.299_300del | p.H100Rfs*14 | Hom | Frameshift | P | AR |
| 326 | *GJB2* | Exon2 | c.299_300del | p.H100Rfs*14 | Hom | Frameshift | P | AR |
| 309 | *GJB2* | Exon2；Exon2 | c.235delC；c.109G>A | p.L79Cfs*3；p.V37I | Het；Het | Frameshift；Missense | P；P | AR |
| 298 | *GJB2* | Exon2 | c.235delC | p.L79Cfs*3 | Hom | Frameshift | P | AR |
| 289 | *GJB2* | Exon2；Exon2 | c.478G>A；c.235delC | p.G160S；p.L79Cfs*3 | Hom；Hom | Missense；Frameshift | U；P | AR |
| 287 | *GJB2* | Exon2；Exon2 | c.299_300del；c.176_191del | p.H100Rfs*14；p.G59Afs*18 | Het；Het | Frameshift；Frameshift | P；P | AR |
| 273 | *GJB2* | Exon2 | c.235delC | p.L79Cfs*3 | Hom | Frameshift | P | AR |
| 320 | *GJB2* | Exon3 | c.235delC | p.L79Cfs*3 | Hom | Frameshift | P | AR |
| 312 | *GJB2* | Exon4 | c.109G>A | p.V37I | Hom | Missense | P | AR |
| 291 | *GJB2* | Exon5 | c.109G>A | p.V37I | Hom | Missense | P | AR |
| 288 | *GJB2* | Exon2；Exon2 | c.299_300del；c.176_191del | p.H100Rfs*14；p.G59Afs*18 | Het；Het | Frameshift；Frameshift | P；P | AR |
| 829 | *GJB2* | Exon2；Exon2 | c.299_300delAT；c.235delC | p.H100Rfs*14；p.L79Cfs*3 | Het；Het | Frameshift；Frameshift | P；P | AR |
| 833 | *GJB2* | Exon2 | c.235delC | p.L79Cfs*3 | Hom | Frameshift | P | AR |
| 845 | *GJB2* | Exon2；Exon2 | c.176_191delGCTGCAAGAACGTGTG；c.299_300delAT | p.G59Afs*18；p.H100Rfs*14 | Het；Het | Frameshift；Frameshift | P；P | AR |
| 863 | *GJB2* | Exon2；Exon2 | c.139G>T；c.551G>C | p.E47X；p.R184P | Het；Het | Nonsense；Missense | P；P | AR |
| 878 | *GJB2* | Exon2 | c.109G>A | p.V37I | Hom | Missense | P | AR |
| 820 | *GJB2* | Exon2；Exon2 | c.508_511dupAACG；c.235delC | p.A171Efs*40；p.L79Cfs*3 | Het；Het | Frameshift；Frameshift | P；P | AR |
| 600 | *GJB2* | Exon2；Exon2 | c.11G>A/c.235delC | p.Gly4Asp；p.Leu79fs | Het；Het | Missense；Missense | U；P | AR |
| 561 | *GJB2* | Exon2；Exon2 | c.139G>T\c.176_191del | p.Glu47*；p.Gly59fs | Het；Het | Nonsense；Missense | U；U | AR |
| 530 | *GJB2* | Exon2；Exon2 | c.11G>A/c.235delC | p.Gly4Asp；p.Leu79fs | Het；Het | Missense；Missense | L；P | AR |
| 643 | *GJB2* | Exon2 | c.109G>A | p.Val37Ile | Hom | Missense | P | AR |
| 680 | *GJB2* | Exon2；Exon2 | c.109G>A；c.235delC | p.Val37Ile；p.Leu79Cysfs | Het；Het | Missense；Frameshift | P；P | AR |
| 686 | *GJB2* | Exon2；Exon2 | c.235delC；c.257C>G | p.Leu79Cysfs；p.Thr86Arg | Het；Het | Missense；Missense | P；P | AR |
| 703 | *GJB2* | Exon2；Exon2 | c.109G>A；c.235delC | p.Val37Ile；p.Leu79Cysfs | Het；Het | Missense；Frameshift | P；P | AR |
| 710 | *GJB2* | Exon2 | c.109G>A | p.Val37Ile | Hom | Missense | P | AR |
| 716 | *GJB2* | Exon2 | c.109G>A | p.Val37Ile | Hom | Missense | P | AR |
| 725 | *GJB2* | Exon2；Exon2 | c.235delC；c.176_191del | p.Leu79Cysfs；p.Gly59Alafs | Het；Het | Frameshift；Frameshift | P；P | AR |
| 728 | *GJB2* | Exon2；Exon2 | c.299_300del；c.235delC | p.His100Argfs；p.Leu79Cysfs | Het；Het | Frameshift；Frameshift | P；P | AR |
| 729 | *GJB2* | Exon2 | c.257C>G | p.Thr86Arg | Hom | Missense | P | AR |
| 737 | *GJB2* | Exon2；Exon2 | c.35dupG | p.Val13Cysfs | Hom | Frameshift | P | AR |
| 748 | *GJB2* | Exon2；Exon2 | c.257C>G ； c.235delC | p.Thr86Arg；p.Leu79Cysfs | Het；Het | Missense；Missense | P；P | AR |
| 752 | *GJB2* | Exon2 ；Exon2 | c.230G>A ；c.235delC | p.Trp77*；p.Leu79Cysfs | Het；Het | Nonsense；Frameshift | P；P | AR |
| 753 | *GJB2* | Exon2 | c.109G>A | p.Val37Ile | Hom | Missense | P | AR |
| 754 | *GJB2* | Exon2 ；Exon2 | c.109G>A；c.358_360del | p.Val37Ile；p.Glu120del | Het；Het | Missense；Inframe | P；P | AR |
| 767 | *GJB2* | Exon2 | c.139G>T | p.Glu47* | Hom | Nonsense | P | AR |
| 775 | *GJB2* | Exon2 ；Exon2 | c.109G>A ；c.368C>A | p.Val37Ile；p.Thr123Asn | Het；Het | Missense；Missense | P；U | AR |
| 806 | *GJB2* | Exon2；Exon2 | c.299_300del ；c.235delC | p.His100Argfs ；p.Leu79Cysfs | Het；Het | Frameshift；Frameshift | P；P | AR |
| 818 | *GJB2* | Exon2 ；Exon2 | c.299_300del ；c.235delC | p.His100Argfs；p.Leu79Cysfs | Het；Het | Frameshift；Frameshift | P；P | AR |
| 54 | *SLC26A4* | Exon19；Exon15 | c.2168A>G；c.1707+5G>A | p.H72R；(-) | Het；Het | Missense；Splicing | P；P | AR |
| 146 | *SLC26A4* | Exon8；Exon19 | c.919-2A>G；c.2168A>G | (-)；p.H723R | Het；Het | Splicing；Missense | P；P | AR |
| 141 | *SLC26A4* | Exon5；Exon10 | c.589G>A；c.1174A>T | p.G197R；p.N392Y | Het；Het | Missense；Missense | P；P | AR |
| 185 | *SLC26A4* | UTR3；Exon8 | c.*69C>A；c.919-2A>G | (-)；(-) | Het；Het | UTR3；Splicing | P；P | AR |
| 179 | *SLC26A4* | Exon8 | c.919-2A>G | (-) | Hom | Splicing | P | AR |
| 209 | *SLC26A4* | Exon3；Exon8 | c.281C>T；c.919-2A>G | p.T94I；(-) | Het；Het | Missense；Splicing | P；P | AR |
| 226 | *SLC26A4* | Exon8；Exon19 | c.919-2A>G；c.2176A>G | (-)；p.I726V | Het；Het | Splicing；Missense | P；P | AR |
| 250 | *SLC26A4* | Exon8 | c.919-2A>G | (-) | Hom | Splicing | P | AR |
| 252 | *SLC26A4* | Exon6；Exon19 | c.754T>C；c.2168A>G | p.S252p；p.H723R | Het；Het | Missense；Missense | L；L | AR |
| 253 | *SLC26A4* | Exon8 | c.919-2A>G | (-) | Hom | Splicing | P | AR |
| 264 | *SLC26A4* | Exon10；Exon18 | c.1173C>A；c.2086C>T | p.S391R； p.Q696X | Het；Het | Missense；Missense | P；P | AR |
| 345 | *SLC26A4* | Exon2 | c.86A>G | p.E29G | Hom | Missense | P | AR |
| 349 | *SLC26A4* | Exon10；Exon2-3 | c.1174A>T；CNV | p.N392Y；(-) | Het；Het | Missense；CNV | P；P | AR |
| 409 | *SLC26A4* | Exon8 | c.919-2A>G | (-) | Hom | Splicing | P | AR |
| 446 | *SLC26A4* | Exon12；Exon10 | c.1343_1355dupCGGTCTTGGCAGC；c.1174A>T | p.V453Gfs*19；p.N392Y | Het；Het | Frameshift；Missense | P；P | AR |
| 401 | *SLC26A4* | Exon10；Exon3 | c.1229C>T；c.304+3A>C | p.T410M；(-) | Het；Het | Missense；Splicing | P；P | AR |
| 436 | *SLC26A4* | Exon1-3；Exon10 | CNV；c.1262A>C | (-)；p.Q421P | Het；Het | CNV；Missense | P；P | AR |
| 489 | *SLC26A4* | Exon6 | c.754T>C | p.S252P | Hom | Missense | P | AR |
| 495 | *SLC26A4* | Intron19 | c.2236-25T>A | IVS19 as T-A-25 | Hom | Intronic | U | AR |
| 497 | *SLC26A4* | Exon7；Exon8 | c.916dupG；c.919-2A>G | p.V306Gfs*24；(-) | Het；Het | Frameshift；Splicing | P；P | AR |
| 2 | *SLC26A4* | Exon8；Exon3 | c.919-2A>G；c.281C>T | (-)；p.T94I | Het；Het | Splicing；Missense | P；L | AR |
| 6 | *SLC26A4* | Exon18；Exon4 | c.2086C>T；c.312_322delATATGCCCTAC | p.Q696X；p.Y105Sfs*73 | Het；Het | Nonsense；Frameshift | P；P | AR |
| 846 | *SLC26A4* | Exon8 | c.919-2A>G | (-) | Hom | Splicing | P | AR |
| 371 | *SLC26A4* | Exon8；Exon15 | c.919-2A>G；c.1707+5G>A | (-)；(-) | Het；Het | Splicing；Splicing | P；LP | AR |
| 355 | *SLC26A4* | Exon14；Exon18 | c.1546dupC；c.2086C>T | p.S517Ffs*10；p.Q696X | Het；Het | Frameshift；Nonsense | P；P | AR |
| 301 | *SLC26A4* | Exon4；Exon8 | **c.311_321del；c.919-2A>G** | p.Y105Sfs*73；(-) | Het；Het | Frameshift；Splicing | P；P | AR |
| 294 | *SLC26A4* | Exon8；Exon13 | c.919-2A>G；c.1519delT | (-)；p.L507X | Het；Het | Frameshift；Nonsense | P；P | AR |
| 107 | *SLC26A4* | Exon8 | c.919-2A>G | (-) | Hom | Splicing | P | AR |
| 450 | *SLC26A4* | Exon8，Exon19 | c.919-2A>G；c.2168A>G | (-)；p.H723R | Het；Het | Splicing；Missense | P；P | AR |
| 140 | *SLC26A4* | Exon8；Exon18 | c.919-2A>G；c.2086C>T | (-)；p.Q696X | Het；Het | Splicing；Missense | P；P | AR |
| 32 | *SLC26A4* | Exon8；Exon5 | c.919-2A>G；c.589G>A | (-)；p.G197R | Het；Het | Splicing；Missense | P；P | AR |
| 369 | *SLC26A4* | Exon8；Exon6 | c.919-2A>G；c.716T>A | (-)；p.V239D | Het；Het | Splicing；Missense | P；P | AR |
| 121 | *SLC26A4* | Exon13；Exon8 | c.919-2A>G；c.1520delT | (-)；p.L507X | Het；Het | Splicing；Nonsense | P；P | AR |
| 49 | *SLC26A4* | Exon11 | c.1264-12T>A | (-) | Hom | Intronic | LP | AR |
| 852 | *SLC26A4* | Exon8；Exon19 | c.919-2A>G；c.2168A>G | (-)；p.H723R | Het；Het | Splicing；Missense | P；P | AR |
| 879 | *SLC26A4* | Exon8 | c.919-2A>G | (-) | Hom | Splicing | P | AR |
| 136 | *SLC26A4* | Exon8 | c.919-2A>G | (-) | Hom | Splicing | P | AR |
| 603 | *SLC26A4* | Exon13 | c.1522A>G | p.Thr508Ala | Hom | Missense | LP | AR |
| 623 | *SLC26A4* | Exon15；Intron7 | c.1692dupA/c.919-2A>G | p.Cys565fs；(-) | Het；Het | Frameshift；Splicing | P；P | AR |
| 539 | *SLC26A4* | Exon2；Exon2-3 | c.86A>G；CNV | p.Glu29Gly | Het；Het | Missense；CNV | U | AR |
| 544 | *SLC26A4* | Exon13 | c.1519delT | p.Leu507* | Hom | Nonsense | P | AR |
| 558 | *SLC26A4* | Exon10；Exon2-3 | c.1174A>T；CNV | p.Asn392Tyr；(-) | Het；Het | Missense；CNV | P | AR |
| 505 | *SLC26A4* | Exon3 | c.290T>A | p.Val97Glu | Hom | Missense | U | AR |
| 524 | *SLC26A4* | Exon10；Exon2-3 | c.1174A>T；CNV | p.Asn392Tyr；(-) | Het；Het | Missense；CNV | P | AR |
| 529 | *SLC26A4* | Exon3；Intron7 | c.296C>G；c.919-2A>G | p.Thr99Arg；(-) | Het；Het | Missense；Splicing | LP；P | AR |
| 532 | *SLC26A4* | Intron7；Exon2-3 | c.919-2A>G；CNV | (-)；2-3Exon | Het；Het | Missense；CNV | P | AR |
| 684 | *SLC26A4* | Intron7；Exon10 | c.919-2A>G；c.1174A>T | (-)；p.Asn392Tyr | Het；Het | Splicing；Missense | P；P | AR |
| 694 | *SLC26A4* | Exon10；Intron13 | c.1229C>T；c.1544+5G>A | p.Thr410Met；(-) | Het；Het | Missense；Splicing | P；P | AR |
| 698 | *SLC26A4* | Exon10；Intron13 | c.1229C>T；c.1544+5G>A | p.Thr410Met；(-) | Het；Het | Missense；Splicing | P；P | AR |
| 712 | *SLC26A4* | Exon19；Exon10 | c.2168A>G；c.1262A>C | p.His723Arg；p.Gln421Pro | Het；Het | Missense；Missense | LP；U | AR |
| 756 | *SLC26A4* | Exon14；Intron15 | c.1594A>C；c.1707+5G>A | p.Ser532Arg；(-) | Het；Het | Missense；Splicing | U；P | AR |
| 191 | *MYO15A* | Exon9 | **c.4143-1G>A** | **(-)** | Hom | Splicing | U | AR |
| 9 | *MYO15A* | Exon23；Exon21 | **c.5681T>C；c.6721T>C** | **p.L1894P；p.W2241R** | Het；Het | Missense；Missense | U | AR |
| 18 | *MYO15A* | Exon63 | **c.10350+2T>G** | **(-)** | Hom | Splicing | LP | AR |
| 431 | *MYO15A* | Exon16；Exon63 | **c.5005delC；c.10250_10252delCCT** | p.Q1669Rfs*128；p.S3417del | Het；Het | Frameshift；Inframe | LP；LP | AR |
| 29 | *MYO15A* | Exon63 | **c.10350+2T>G** | **(-)** | Hom | Splicing | LP | AR |
| 274 | *MYO15A* | Exon15；Exon25 | c.4823C>A；c.5964+3G>A | p.A1608E；(-) | Het；Het | Missense；Splicing | LP；LP | AR |
| 491 | *MYO15A* | Exon59；Exon64 | **c.9787+1G>A；c.10419_10423del** | **(-)；p.S3474Pfs*42** | Het；Het | Splicing；Frameshift | LP；LP | AR |
| 365 | *MYO15A* | Exon63；Exon64 | c.10245_10247delCTC；c.10419_10423del | p.3415_3416delGSinsG；p.S3474Pfs*42 | Het；Het | Inframe ；Frameshift | U；LP | AR |
| 353 | *MYO15A* | Exon27；Exon55 | c.6177+1G>T；c.9385C>T | (-)；p.Q3129X | Het；Het | Splicing；Nonsense | P；LP | AR |
| 341 | *MYO15A* | Exon63 | **c.10350+2T>G** | **(-)** | Hom | Splicing | LP | AR |
| 337 | *MYO15A* | Exon32；Exon63 | c.6956+9C>G；c.10245_10247delCTC | (-)；p.3415_3416delGSinsG | Het；Het | Splicing；Inframe | LP；U | AR |
| 278 | *MYO15A* | Exon64 | **c.10419_10423del** | **p.S3474Pfs*42** | Hom | Frameshift | LP | AR |
| 270 | *MYO15A* | Exon9；Exon9 | **c.4143-1G>A** | **(-)** | Hom | Splicing | LP | AR |
| 281 | *MYO15A* | Exon56 | c.9478C>T | p.L3160F | Hom | Missense | U | AR |
| 599 | *MYO15A* | Exon16；Exon64 | **c.4970T>A；c.10484dupT** | **p.Ile1657Asn；p.Glu3496fs** | Het；Het | Missense；Frameshift | U；P | AR |
| 609 | *MYO15A* | Intron63 | **c.10350+2T>G** | **p.?** | Hom | Splicing | P | AR |
| 559 | *MYO15A* | Exon14；Intron | c.4760T>G；c.9690+1G>A | p.Leu1587Arg；(-) | Het；Het | Missense；Splicing | U；P | AR |
| 526 | *MYO15A* | Exon23；Intron43 | **c.5693G>A；c.8149-11T>A** | **p.Arg1898Gln；(-)** | Het；Het | Missense；Splicing | U；U | AR |
| 602 | *MYO15A* | Intron63 | **c.10350+2T>G** | **(-)** | Hom | Splicing | P | AR |
| 685 | *MYO15A* | Intron58；Exon17 | c.9690+1G>A；c.5023T>C | (-)；p.Phe1675Leu | Het；Het | Splicing；Missense | P；U | AR |
| 791 | *MYO15A* | Exon63 | c.10258_10260del | p.Phe3420del | Hom | Inframe | LP | AR |
| 795 | *MYO15A* | Exon50 | **c.8944C>T** | **p.Gln2982*** | Hom | Nonsense | LP | AR |
| 234 | *MYO7A* | Exon29 | **c.3633delC** | **p.L1212Cfs*20** | Het | Frameshift | P | AD |
| 416 | *MYO7A* | Exon32；Exon41 | c.4254delC；c.5648G>T | p.D1419Tfs*7；p.R1883L | Het；Het | Frameshift；Missense | P；LP | AR |
| 249 | *MYO7A* | Exon8 | **c.765C>A** | **p.F255L** | Hom | Missense | U | AR |
| 305 | *MYO7A* | Exon32 | **c.4237G>A** | **p.V1413M** | Het | Missense | U | AD |
| 821 | *MYO7A* | Exon4；Exon35 | c.160A>G；c.4757A>G | p.T54A；p.N1586S | Het；Het | Missense；Missense | U；U | AR |
| 476 | *MYO7A* | Exon27 | **c.3494C>T** | **p.P1165L** | Het | Missense | U | AD |
| 382 | *MYO7A* | Exon35 | **c.4757A>G** | **p.N1586S** | Het | Missense | U | AD |
| 627 | *MYO7A* | Exon7；Exon47 | **c.638T>A；c.6367C>T** | **p.Phe213Tyr；p.Pro2123Ser** | Het；Het | Missense；Missense | U；U | AR |
| 523 | *MYO7A* | Intron30 | **c.3924+8C>A** | **p.?** | Het | Splicing | U | AD |
| 652 | *MYO7A* | Exon14 | **c.1564A>T** | **p.Thr522Ser** | Het | Missense | U | AD |
| 679 | *MYO7A* | Exon43 | c.5880_5882del | p.F1963del | Hom | Inframe | LP | AR |
| 683 | *MYO7A* | Exon31 | c.3935T>C | p.Leu1312Pro | Hom | Missense | U | AR |
| 688 | *MYO7A* | Exon39 | **c.5374G>A** | **p.Val1792Ile** | Het | Missense | U | AD |
| 718 | *MYO7A* | Intron29 | **c.3750+4C>T** | **(-)** | Het | Intronic | U | AD |
| 723 | *MYO7A* | Exon11 | c.1142C>T | p.Thr381Met | Het | Missense | U | AD |
| 731 | *MYO7A* | Exon25 | **c.3158delC** | **p.Pro1053Leufs** | Hom | Frameshift | LP | AR |
| 741 | *MYO7A* | Exon35 | **c.4619C>T** | **p.Ala1540Val** | Het | Missense | U | AD |
| 759 | *MYO7A* | Exon8；Exon40 | **c.765C>A ；c.5518T>C** | **p.Phe255Leu；p.Cys1840Arg** | Het；Het | Missense；Missense | U；U | AR |
| 764 | *MYO7A* | Exon6 ； Exon31 | c.487G>A ；c.3935T>C | p.Gly163Arg；p.Leu1312Pro | Het；Het | Missense；Missense | U；U | AR |
| 807 | *MYO7A* | Exon7 | c.634C>T | p.Arg212Cys | Het | Frameshift | P | AD |
| 808 | *MYO7A* | Exon8 ；Exon40 | **c.765C>A ；c.5518T>C** | **p.Phe255Leu；p.Cys1840Arg** | Het；Het | Missense；Missense | U；U | AR |
| 388 | *CDH23* | Exon37-50 | **CNV** | **(-)** | Hom | CNV | P | AR |
| 485 | *CDH23* | Exon27；Exon48 | c.3262G>A；c.6911G>A | p.V1088M；p.R2304Q | Het；Het | Missense；Missense | U；U | AR |
| 475 | *CDH23* | Exon9；Exon24 | **c.805C>T；c.2866G>A** | p.R269W；p.E956K | Het；Het | Missense；Missense | U；LP | AR |
| 194 | *CDH23* | Exon46；Exon46 | c.6604G>A；c.6504T>A | p.D2202N；p.N2168K | Het；Het | Missense；Missense | LP；U | AR |
| 646 | *CDH23* | Exon44；Exon31 | c.5985C>A；c.3902T>A | p.Tyr1995*；p.Ile1301Asn | Het；Het | Nonsense；Missense | LP；U | AR |
| 687 | *CDH23* | Exon46 | **c.6649A>G** | **p.Lys2217Glu** | Hom | Missense | U | AR |
| 691 | *CDH23* | Exon46；Intron2 | **c.6654C>A；c.68-3C>T** | p.Asp2218Glu；p.？ | Het；Het | Missense；Intronic | U；U | AR |
| 702 | *CDH23* | Exon36；Exon16 | **c.4783G>A；c.1765G>A** | **p.Glu1595Lys；p.Asp589Asn** | Het；Het | Missense；Missense | U；U | AR |
| 711 | *CDH23* | Exon27；Exon48 | c.3262G>A；c.6911G>A | p.Val1088Met；p.Arg2304Gln | Het；Het | Missense；Missense | U；U | AR |
| 727 | *CDH23* | Exon68；Exon47 | **c.9860G>A；c.6809G>A** | **p.Gly3287Asp；p.Arg2270His** | Het；Het | Missense；Missense | U；U | AR |
| 734 | *CDH23* | Exon8 ；Exon35 | **c.683A>G；c.4562A>G** | p.Asp228Gly ；p.Asn1521Ser | Het；Het | Missense；Missense | U；U | AR |
| 735 | *CDH23* | Exon35；Exon36 | c.4562A>G ； c.4783G>A | p.Asn1521Ser；p.Glu1595Lys | Het；Het | Missense；Missense | U；U | AR |
| 738 | *CDH23* | Exon47 | **c.6809G>A** | **p.Arg2270His** | Hom | Missense | U | AR |
| 804 | *CDH23* | Exon31 ；Exon47 | **c.4054G>A；c.6809G>A** | **p.Ala1352Thr；p.Arg2270His** | Het；Het | Missense；Missense | U；U | AR |
| 158 | *MITF* | Exon1 | c.37C>T | p.R13X | Het | Nonsense | LP | AD |
| 168 | *MITF* | Exon7 | c.641_643delGAA | p.214_215delRRinsR | Het | Inframe | P | AD |
| 167 | *MITF* | Exon3-10 | CNV | (-) | Het | CNV | P | AD |
| 381 | *MITF* | Exon4 | **c.419delC** | **p.P140fs*15** | Het | Frameshift | L | AD |
| 501 | *MITF* | Exon9 | **c.1024delinsAT** | **p.D342Ifs*79** | Het | Frameshift | P | AD |
| 479 | *MITF* | Exon4 | **c.434_435insAA** | **p.N146Kfs*10** | Het | Frameshift | L | AD |
| 592 | *MITF* | Exon8 | **c.796G>T** | **p.Glu266*** | Het | Nonsense | P | AD |
| 610 | *MITF* | Exon7 | **c.672_673dupTA** | **p.Lys225fs** | Het | Frameshift | P | AD |
| 632 | *MITF* | Exon9 | **c.871C>T** | **p.Gln291*** | Het | Nonsense | P | AD |
| 549 | *MITF* | Exon7 | **c.650G>A** | **p.Arg217Lys** | Het | Missense | U | AD |
| 520 | *MITF* | Exon7 | **c.647_648delinsTT** | **p.Arg216Ile** | Het | Missense | U | AD |
| 642 | *MITF* | Intron6 | **c.634+1G>A** | **(-)** | Het | Splicing | LP | AD |
| 717 | *MITF* | Exon9 | c.1060C>A | p.Leu354Ile | Het | Missense | U | AD |
| 217 | *TMC1* | Exon7 | **c.107delA** | **p.E36Gfs*3** | Hom | Frameshift | LP | AR |
| 216 | *TMC1* | Exon13 | c.797T>C | p.I266T | Het | Missense | LP | AD |
| 421 | *TMC1* | Exon13；Exon19 | c.884+2_884+3insT；c.1744T>C | splicing；vp.F582L | Het；Het | Splicing；Missense | LP；U | AR |
| 474 | *TMC1* | Exon18 | **c.1672dupT** | **p.C558Lfs*66** | Het | Frameshift | L | AD |
| 299 | *TMC1* | Exon20 | **c.1953delC** | **p.L652Cfs*3** | Hom | Frameshift | L | AR |
| 612 | *TMC1* | Intron9 | **c.453+5G>A** | **(-)** | Hom | Splicing | U | AR |
| 631 | *TMC1* | Exon15 | **c.1110C>A** | **p.Phe370Leu** | Het | Missense | U | AD |
| 546 | *TMC1* | Exon18 | **c.1672dupT** | **p.Cys558fs** | Hom | Frameshift | P | AD |
| 567 | *TMC1* | Exon18 | **c.1672dupT** | **p.Cys558fs** | Hom | Frameshift | P | AR |
| 536 | *TMC1* | Exon12 | **c.697G>A** | **p.Glu233Lys** | Het | Missense | U | AD |
| 706 | *TMC1* | Exon16 | c.1333C>T | p.Arg445Cys | Hom | Missense | U | AR |
| 740 | *TMC1* | Intron13 | **c.884+2dupT** | **(-)** | Het | Splicing | U | AD |
| 778 | *TMC1* | Exon7 | **c.196G>A** | **p.Ala66Thr** | Het | Missense | U | AD |
| 208 | *USH2A* | Exon57；Exon28 | **c.11053T>C；c.5608C>T** | p.W3685R；p.R1870W | Het；Het | Missense；Missense | U；LP | AR |
| 359 | *USH2A* | Exon21；Exon2 | c.4616C>T；c.206G>T | p.T1539I；p.S69I | Het；Het | Missense；Missense | LP；U | AR |
| 638 | *USH2A* | Exon2 | c.99_100insT | p.Arg34fs | Hom | Frameshift | P | AR |
| 542 | *USH2A* | Intron39；Exon6 | c.7451+3G>C；c.1105G>A | (-)；p.Val369Met | Het；Het | Missense；Splicing | U；U | AR |
| 564 | *USH2A* | Exon70；Exon13 | c.15233C>G；c.2802T>G | p.Pro5078Arg；p.Cys934Trp | Het；Het | Missense；Missense | U；U | AR |
| 644 | *USH2A* | Exon37；Exon21 | c.6998T>C；c.4616C>T | p.Val2333Ala；p.Thr1539Ile | Het；Het | Missense；Missense | U；U | AR |
| 648 | *USH2A* | Exon47；Intron5 | c.9259G>A；c.848+5G>C | p.Val3087Ile；(-) | Het；Het | Missense；Intronic | U；U | AR |
| 676 | *USH2A* | Intron54；Intron5 | **c.10740+7G>A；c.848+5G>C** | **(-)；(-)** | Het；Het | Intronic；Intronic | U；U | AR |
| 758 | *USH2A* | Exon37 ；Exon46 | c.7068T>G；c.9244A>G | p.Asn2356Lys；p.Ile3082Val | Het；Het | Missense；Missense | U；U | AR |
| 811 | *USH2A* | Exon66；Exon52 | **c.14567A>G ；c.10312G>A** | **p.Asn4856Ser ；p.Ala3438Thr** | Het；Het | Missense；Missense | U；U | AR |
| 812 | *USH2A* | Intron5 ；Exon19 | c.848+5G>C；c.4241C>T | p.?；p.Ala1414Val | Het；Het | Intronic；Missense | U；U | AR |
| 817 | *USH2A* | Exon67 ；Exon30 | **c.14600A>T ；c.6001C>T** | p.His4867Leu；p.Arg2001Cys | Het；Het | Missense；Missense | U；U | AR |
| 819 | *USH2A* | Exon67；Exon30 | **c.14600A>T ；c.6001C>T** | p.His4867Leu；p.Arg2001Cys | Het；Het | Missense；Missense | U；U | AR |
| 710 | *WFS1* | Exon8 | c.1514G>C | p.C505S | Het | Missense | LP | AD |
| 496 | *WFS1* | Exon5 | c.535G>A | p.A179T | Het | Missense | U | AD |
| 490 | *WFS1* | Exon8；Exon8 | c.1820C>T；c.2603G>A | p.P607L；p.R868H | Het；Het | Missense；Missense | LP；LP | AR |
| 443 | *WFS1* | Exon8 | c.2051C>T | p.A684V | Het | Missense | LP | AD |
| 275 | *WFS1* | Exon8 | c.1820C>T | p.P607L | Het | Missense | LP | AD |
| 271 | *WFS1* | Exon8 | c.2051C>T | p.A684V | Het | Missense | LP | AD |
| 511 | *WFS1* | Exon8 | c.2051C>T | p.Ala684Val | Het | Missense | LP | AD |
| 667 | *WFS1* | Exon8  Exon8 | c.1820C>T  c.2603G>A | p.Pro607Leu；p.Arg868His | Het；Het | Missense；Missense | U；U | AR |
| 695 | *WFS1* | Exon7 | **c.818A>C** | **p.Glu273Ala** | Het | Missense | U | AD |
| 763 | *WFS1* | Exon8 ；Exon8 | c.1820C>T ；c.2603G>A | p.Pro607Leu；p.Arg868His | Het；Het | Missense；Missense | U；U | AR |
| 782 | *WFS1* | Exon8 | c.1309G>A | p.Gly437Ser | Het | Missense | U | AD |
| 106 | *SOX10* | Exon2 | **c.127C>T** | **p.R43X** | Het | Nonsense | LP | AD |
| 163 | *SOX10* | Exon2 | c.346C>G | p.Q116E | Het | Missense | P | AD |
| 172 | *SOX10* | Exon4 | c.698-2A>C | (-) | Het | Splicing | P | AD |
| 269 | *SOX10* | Exon2 | **c.23C>A** | **p.S8X** | Het | Nonsense | LP | AD |
| 386 | *SOX10* | Exon2 | **c.178delG** | **p.D60fs*49** | Het | Frameshift | P | AD |
| 498 | *SOX10* | Exon3 | c.506delC | p.P169Rfs*117 | Het | Frameshift | P | AD |
| 871 | *SOX10* | Exon2 | **c.77_104delGGAGCGCGCCCTCGCTAGGGCCCGACGG** | **p.G26Afs*74** | Het | Frameshift | LP | AD |
| 595 | *SOX10* | Exon2 | c.255G>A | p.Trp85* | Het | Nonsense | P | AD |
| 569 | *SOX10* | Exon2 | c.398A>G | p.Glu133Gly | Het | Missense | U | AD |
| 514 | *SOX10* | Exon3 | **c.448A>G** | **p.Lys150Glu** | Het | Missense | U | AD |
| 30 | *COL11A2* | Exon4；Exon5 | **c.628A>G；c.836C>A** | **p.I210V；p.P279H** | Het；Het | Missense；Missense | U | AR |
| 247 | *COL11A2* | Exon14 | **c.1475T>C** | **p.M492T** | Het | Missense | U | AD |
| 665 | *COL11A2* | Exon2  Intron65 | **c.230C>A**  **c.5071-5T>G** | **p.Pro77Gln；(-)** | Het；Het | Missense；Missense | U；U | AR |
| 681 | *COL11A2* | Exon5 | c.688G>T | p.Gly230Trp | Het | Missense | U | AD |
| 701 | *COL11A2* | Intron24 | **c.2017-5T>G** | **(-)** | Het | Intronic | U | AD |
| 10 | *COL11A2* | Exon5 | c.688G>T | p.Gly230Trp | Het | Missense | U | AD |
| 781 | *COL11A2* | Intron65；Exon2 | **c.5071-5T>G；c.230C>A** | **(-)；p.Pro77Gln** | Het；Het | Intronic；Missense | U；U | AR |
| 786 | *COL11A2* | Exon5 | c.688G>T | p.Gly230Trp | Het | Missense | U | AD |
| 799 | *COL11A2* | Exon51 | **c.3725C>T** | **p.Ser1242Leu** | Het | Missense | U | AD |
| 802 | *COL11A2* | Exon30 | **c.2336C>T** | **p.Pro779Leu** | Het | Missense | U | AD |
| 452 | *OTOF* | Exon30；Exon3 | **c.3748C>T；c.157G>A** | p.R1250C；p.A53T | Het；Het | Missense；Missense | U；U | AR |
| 60 | *OTOF* | Exon30；Exon12 | **c.3864+8G>A；c.1194T>A** | splicing；p.D398E | Het；Het | Splicing；Missense | U；U | AR |
| 591 | *OTOF* | Exon44；Exon40 | c.5647C>T；c.5000C>A | p.Gln1883*；p.Ala1667Asp | Het；Het | Nonsense；Missense | P；L | AR |
| 630 | *OTOF* | Exon44 | **c.5668T>C** | **p.Trp1890Arg** | Hom | Missense | U | AR |
| 662 | *OTOF* | Intron32；Intron46 | c.4023+1G>A；c.6012-10C>T | (-)；(-) | Het；Het | Splicing；Splicing | LP；U | AR |
| 689 | *OTOF* | Exon16 | **c.1829delT** | **p.Phe610Serfs** | Hom | Frameshift | LP | AR |
| 720 | *OTOF* | Exon38 | **c.4791delC** | **p.Tyr1598Thrfs** | Hom | Frameshift | P | AR |
| 750 | *OTOF* | Exon38 | **c.4791delC** | **p.Tyr1598Thrfs** | Hom | Frameshift | P | AR |
| 769 | *OTOF* | Intron18 | c.2215-1G>C | (-) | Hom | Splicing | LP | AR |
| 803 | *OTOF* | Intron18 | c.2215-1G>C | (-) | Hom | Splicing | LP | AR |
| 260 | *PTPN11* | Exon13 | c.1510A>G | p.M504V | Het | Missense | LP | AD |
| 434 | *PTPN11* | Exon13 | c.1502G>A | p.R501K | Het | Missense | LP | AD |
| 484 | *PTPN11* | Exon1 | c.5C>T | p.T2I | Het | Missense | LP | AD |
| 335 | *PTPN11* | Exon7 | c.836A>G | p.Y279C | Het | Missense | LP | AD |
| 861 | *PTPN11* | Exon4 | c.417G>C | p.E139D | Het | Missense | P | AD |
| 590 | *PTPN11* | Exon13 | c.1510A>G | p.Met504Val | Het | Frameshift | LP | AD |
| 604 | *PTPN11* | Exon1 | c.5C>T | p.Thr2Ile | Het | Missense | U | AD |
| 518 | *PTPN11* | Exon7 | c.836A>G | p.Tyr279Cys | Het | Missense | P | AD |
| 181 | *OTOG* | Exon31；Exon49 | **c.3805A>G；c.7991A>G** | **p.M1269V；p.D2664G** | Het；Het | Missense；Missense | U；U | AR |
| 492 | *OTOG* | Exon8；Exon51 | c.919G>A；c.8012-6A>T | p.V307M；(-) | Het；Het | Missense；Splicing | U；U | AR |
| 647 | *OTOG* | Exon19；Exon35 | **c.2438A>G；c.6017C>T** | **p.Asp813Gly；p.Ala2006Val** | Het；Het | Missense；Missense | U；U | AR |
| 700 | *OTOG* | Exon35；Exon39 | **c.6017C>T；c.6737A>G** | **p.Ala2006Val；p.His2246Arg** | Het；Het | Missense；Missense | U；U | AR |
| 713 | *OTOG* | Exon7；Exon33 | **c.819G>T；c.4228G>A** | **p.Met273Ile；p.Glu1410Lys** | Het；Het | Missense；Missense | U；U | AR |
| 755 | *OTOG* | Exon35 ；Exon39 | **c.6017C>T；c.6737A>G** | **p.Ala2006Val ；p.His2246Arg** | Het；Het | Missense；Missense | U；U | AR |
| 231 | *TNC* | Exon12 | **c.3637G>A** | **p.V1213I** | Het | Missense | U | AD |
| 656 | *TNC* | Exon7 | **c.2491G>A** | **p.Asp831Asn** | Het | Nonsense | U | AD |
| 743 | *TNC* | Exon2 | **c.434G>A** | **p.Gly145Asp** | Het | Missense | U | AD |
| 745 | *TNC* | Exon16 | **c.4593_4596del** | **p.Leu1532Lysfs** | Het | Frameshift | LP | AD |
| 761 | *TNC* | Exon4 | **c.1942C>T** | **p.Arg648Trp** | Het | Missense | U | AD |
| 784 | *TNC* | Exon8 | **c.2813T>C** | **p.Val938Ala** | Het | Missense | U | AD |
| 160 | *EYA1* | Exon14；Exon6 | c.1255delT | p.C419Vfs*12 | Het | Frameshift | P | AD |
| 176 | *EYA1* | Exon8-18 | CNV | (-) | Het | CNV | P | AD |
| 212 | *EYA1* | Exon18 | **c.1774delC** | **p.L592Cfs*47** | Het | Frameshift | L | AD |
| 461 | *EYA1* | Exon8 | **c.639+3_639+6delAATT** | **(-)** | Het | Splicing | U | AD |
| 265 | *EYA1* | Exon9 | c.679G>A | p.A227T | Het | Missense | U | AD |
| 225 | *GJB3* | Exon2 | c.474G>A | p.M158I | Het | Missense | LP | AD |
| 658 | *GJB3* | Exon2 | c.580G>A | p.Ala194Thr | Het | Missense | U | AD |
| 671 | *GJB3* | Exon2 | c.538C>T | p.Arg180* | Het | Nonsense | U | AD |
| 719 | *GJB3* | Exon2 | c.250G>A | p.Val84Ile | Het | Missense | LP | AD |
| 721 | *GJB3* | Exon2 | c.250G>A | p.Val84Ile | Het | Missense | LP | AD |
| 596 | *TECTA* | Exon9 | **c.2924G>A** | **p.Arg975Gln** | Het | Missense | U | AD |
| 668 | *TECTA* | Exon13 | **c.4441A>G** | **p.Ser1481Gly** | Het | Missense | U | AD |
| 771 | *TECTA* | Exon10 ；Exon10 | c.3511G>A；c.3149C>A | p.Val1171Met ；p.Thr1050Asn | Het；Het | Missense；Missense | U；U | AR |
| 790 | *TECTA* | Exon18 | **c.5678A>G** | **p.Asn1893Ser** | Het | Missense | U | AD |
| 814 | *TECTA* | Exon13 | **c.4315C>A** | **p.Leu1439Ile** | Het | Missense | U | AD |
| 314 | *BDP1* | Exon39；Exon14 | c.7873T>G；c.2005G>A | p.X2625E；p.E669K | Het；Het | Star/Stop Loss；Missense | LP；U | AR |
| 666 | *BDP1* | Exon2；Exon35 | **c.282A>T；c.7189T>G** | **p.Arg94Ser；p.Tyr2397Asp** | Het；Het | Missense；Missense | U；U | AR |
| 766 | *BDP1* | Exon2；Exon35 | **c.282A>T ；c.7189T>G** | **p.Arg94Ser；p.Tyr2397Asp** | Het；Het | Missense；Missense | U；U | AR |
| 805 | *BDP1* | Exon2；Exon35 | **c.282A>T ；c.7189T>G** | **p.Arg94Ser；p.Tyr2397Asp** | Het；Het | Missense；Missense | U；U | AR |
| 153 | *PAX3* | Exon3 | **c.420_424delCGCGGinsTTAC** | **p.A141Yfs** | Het | Frameshift | LP | AD |
| 155 | *PAX3* | Exon8 | **c.1261C>A** | **p.P421T** | Het | Missense | LP | AD |
| 266 | *PAX3* | Exon6 | **c.830_854del** | **p.Q277Lfs*96** | Het | Frameshift | LP | AD |
| 794 | *PAX3* | Exon5 | **c.602C>G** | **p.Ser201*** | Het | Nonsense | LP | AD |
| 413 | *USH1C* | Exon5 | c.388-1G>A | (-) | Hom | Splicing | LP | AR |
| 483 | *USH1C* | Exon5 | c.388-1G>A | (-) | Hom | Splicing | P | AR |
| 280 | *USH1C* | Exon19 | **c.1527delT** | **p.I509Mfs*2** | Hom | Frameshift | LP | AR |
| 562 | *USH1C* | Exon5 | c.434G>A | p.Cys145Tyr | Hom | Missense | U | AR |
| 373 | *PTPRQ* | Exon37 | **c.5695G>T** | **p.E1899X** | Hom | Nonsense | LP | AR |
| 487 | *PTPRQ* | Exon33 | c.5426+1G>A | (-) | Hom | Splicing | P | AR |
| 292 | *PTPRQ* | Exon33 | **c.5426+1G>C** | **(-)** | Hom | Splicing | P | AR |
| 324 | *PTPRQ* | Exon4；Exon30 | **c.523+9T>G；c.5124G>A** | **(-)；p.M1708I** | Het；Het | Splicing；Missense | U；U | AR |
| 346 | *TRIOBP* | Exon7 | c.964G>T | p.A322S | Hom | Missense | U | AR |
| 554 | *TRIOBP* | Exon7；Exon7 | **c.2176C>T\c.3643delC** | **p.Arg726*；p.Gln1215fs** | Het；Het | Nonsense；Missense | P；P | AR |
| 765 | *TRIOBP* | Exon7 ；Intron17 | **c.951G>T ；c.6325-9G>A** | **p.Glu317Asp ； p.?** | Het；Het | Missense；Intronic | U；U | AR |
| 785 | *TRIOBP* | Exon7 ；Exon7 | c.964G>T；c.3671C>T | p.Ala322Ser)；p.Pro1224Leu | Het；Het | Missense；Missense | U；U | AR |
| 296 | *MYO1A* | Exon10 | **c.829G>A** | **p.V277M** | Het | Missense | U | AD |
| 221 | *MYO1A* | Exon10 | **c.829G>A** | **p.V277M** | Het | Missense | U | AD |
| 568 | *MYO1A* | Exon12 | **c.1061_1062del** | **p.Phe354*** | Het | Nonsense | U | AD |
| 510 | *MYO1A* | Exon11 | **c.935_937del** | **p.Glu312del** | Het | Missense | U | AD |
| 354 | *PCDH15* | Exon35；Exon33 | **c.5135C>T；c.5432T>C** | **p.A1712V；p.L1811P** | Het；Het | Missense；Missense | U；U | AR |
| 724 | *PCDH15* | Intron16 | **c.1997+1G>T** | **(-)** | Hom | Splicing | LP | AR |
| 772 | *PCDH15* | Intron16 | **c.1997+1G>T** | **(-)** | Hom | Splicing | LP | AR |
| 810 | *PCDH15* | Exon18 | **c.2138A>G** | **p.Asn713Ser** | Hom | Missense | U | AR |
| 692 | *WHRN* | Exon6；Exon9 | **c.1352G>A；c.2044C>T** | **p.Gly451Asp；p.Arg682Trp** | Het；Het | Missense；Missense | U；U | AR |
| 714 | *WHRN* | Exon | **c.1352G>A** | **p.Gly451Asp** | Hom | Missense | U | AR |
| 788 | *WHRN* | Intron8；Exon6 | **c.1699-7T>C；c.1352G>A** | **(-)；p.Gly451Asp** | Het；Het | Intronic；Missense | U；U | AR |
| 792 | *WHRN* | Exon6 ；Exon9 | **c.1352G>A；c.2044C>T** | **p.Gly451Asp ；p.Arg682Trp** | Het；Het | Missense；Missense | U；U | AR |
| 362 | *DMXL2* | Exon17 | **c.2778A>T** | **p.E926D** | Het | Missense | U | AD |
| 779 | *DMXL2* | Exon12 | **c.1730C>T** | **p.Thr577Met** | Het | Missense | U | AD |
| 809 | *DMXL2* | Exon9 | **c.1018G>A** | **p.Ala340Thr** | Het | Missense | U | AD |
| 470 | *MYH9* | Exon10 | **c.1108+7C>T** | **splicing** | Het | Splicing | U | AD |
| 41 | *MYH9* | Exon11 | **c.1201G>A** | **p.V401I** | Het | Missense | U | AD |
| 538 | *MYH9* | Intron3 | **c.490+9C>A** | **(-)** | Het | Splicing | U | AD |
| 469 | *LHFPL5* | Exon1 | **c.187G>C** | **p.G63R** | Hom | Missense | U | AR |
| 488 | *LHFPL5* | Exon1 | **c.187G>C** | **p.G63R** | Hom | Missense | U | AR |
| 762 | *LHFPL5* | Exon1 | c.380A>G | p.Tyr127Cys | Hom | Missense | U | AR |
| 442 | *MCM2* | Exon4 | **c.436C>T** | **p.R146C** | Het | Missense | U | AD |
| 308 | *MCM2* | Exon14 | **c.2379G>A** | **p.M793I** | Het | Missense | U | AD |
| 744 | *MCM2* | Exon14 | **c.2379G>A** | **p.Met793Ile** | Het | Missense | U | AD |
| 347 | *TBC1D24* | Exon2 | **c.755A>G** | **p.H252R** | Het | Missense | U | AD |
| 547 | *TBC1D24* | Exon2 | **c.469C>T** | **p.Arg157Cys** | Het | Missense | U | AD |
| 672 | *TBC1D24* | Exon8 | **c.1570C>T** | **p.Arg524Trp** | Het | Missense | U | AD |
| 594 | *COL11A1* | Exon60 | **c.4501G>A** | **p.Gly1501Ser** | Het | Missense | U | AD |
| 655 | *COL11A1* | Exon4 | **c.611C>T** | **p.Thr204Met** | Het | Missense | U | AD |
| 678 | *COL11A1* | Exon59 | **c.4363C>T** | **p.Pro1455Ser** | Het | Missense | U | AD |
| 525 | *LOXHD1* | Exon28；Exon4 | **c.4282C>T；c.441C>T** | **p.Arg1428*；p.Ser147=** | Het；Het | Nonsense；Synonymous | P；U | AR |
| 527 | *LOXHD1* | Exon38；Exon10 | **c.5888delG；c.1420G>T** | **p.Gly1963fs；p.Glu474*** | Het；Het | Frameshift；Nonsense | P；P | AR |
| 768 | *LOXHD1* | Exon21 ；Exon31 | c.3281A>G ；c.4814T>C | p.Asp1094Gly；p.Met1605Thr | Het ； Het | Missense；Missense | U；U | AR |
| 534 | *SLC17A8* | Exon2 | c.161C>T | p.Pro54Leu | Het | Missense | U | AD |
| 770 | *SLC17A8* | Exon9 | c.1120G>T | p.Ala374Ser | Het | Missense | U | AD |
| 787 | *SLC17A8* | Exon9 | c.1120G>T | p.Ala374Ser | Het | Missense | U | AD |
| 670 | *TRRAP* | Exon49 | **c.7241G>C** | **p.Gly2414Ala** | Het | Missense | U | AD |
| 736 | *TRRAP* | Exon44 | **c.6452C>T** | **p.Thr2151Met** | Het | Missense | U | AD |
| 801 | *TRRAP* | Intron36 | c.5259+4A>C | (-) | Het | Missense | U | AD |
| 205 | *FGFR3* | Exon18 | **c.2297C>T** | **p.P766L** | Het | Missense | LP | AD |
| 533 | *FGFR3* | Exon8 | **c.952G>C** | **p.Asp318His** | Het | Missense | U | AD |
| 218 | *MT-RNR1* | - | chrM-1555A>G | - | - | - | - | Mi |
| 545 | *MT-RNR1* | - | m.961delTinsC | - | - | - | - | Mi |
| 327 | *GATA3* | Exon5 | **c.978_979del** | **p.W329Efs*23** | Het | Frameshift | P | AD |
| 243 | *GATA3* | Exon2 | c.59A>G | p.N20S | Het | Missense | U | AD |
| 412 | *SALL4* | Exon4 | **c.3060delG** | **p.Q1020Hfs*57** | Het | Frameshift | LP | AD |
| 48 | *SALL4* | Exon2 | **c.2383G>A** | **p.A795T** | Het | Missense | U | AD |
| 860 | *POU3F4* | Exon1 | **c.101_104dupCTCA** | **p.Q35Hfs*8** | hemi | Frameshift | LP | XLR |
| 117 | *POU3F4* | Exon1 | c.683C>T | p.S228L | hemi | Missense | LP | XLR |
| 130 | *CHD7* | Exon33 | c.6955C>T | p.R2319C | Het | Missense | P | AD |
| 375 | *CHD7* | Exon2 | c.120A>C | p.Q40H | Het | Missense | U | AD |
| 478 | *TSHZ1* | Exon2 | **c.985G>A** | **p.V329M** | Het | Missense | U | AD |
| 321 | *TSHZ1* | Exon2 | **c.929C>T** | **p.A310V** | Het | Missense | U | AD |
| 480 | *MYO6* | Exon28 | c.3057T>G | p.S1019R | Het | Missense | U | AD |
| 303 | *MYO6* | Exon23 | **c.2374C>T** | **p.R792C** | Het | Missense | U | AD |
| 637 | *COL4A6* | Exon31 | **c.3089G>A** | **p.Gly1030Asp** | hemi | Missense | U | XLR |
| 708 | *COL4A6* | Exon23 | **c.1925C>T** | **p.Pro642Leu** | hemi | Missense | U | XLR |
| 657 | *USH1G* | Exon2 | **c.424G>T** | **p.Glu142*** | Hom | Nonsense | LP | AR |
| 661 | *USH1G* | Exon2 | **c.424G>T** | **p.Glu142*** | Hom | Nonsense | LP | AR |
| 715 | *DIAPH3* | Exon25 | c.3124C>T | p.Arg1042Cys | Het | Missense | U | AD |
| 816 | *DIAPH3* | Exon28 | **c.3345delA** | **p.Ser1117Hisfs** | Het | Frameshift | U | AD |
| 751 | *EYA4* | Exon9 | **c.721C>T** | **p.Pro241Ser** | Het | Missense | U | AD |
| 773 | *EYA4* | Exon4 | **c.189_190insAAA** | **p.Val63_Thr64insLys** | Het | Indel-Inframe | U | AD |
| 757 | *DIAPH1* | Exon03 | **c.200C>T** | **p.Ala67Val** | Het | Missense | U | AD |
| 776 | *DIAPH1* | Exon3 | **c.200C>T** | **p.Ala67Val** | Het | Missense | U | AD |
| 400 | *ACTG1* | Exon4 | c.548G>A | p.R183Q | Het | Missense | LP | AD |
| 13 | *ACTB* | Exon4 | c.547C>T | p.R183W | Het | Missense | LP | AD |
| 853 | *ANKRD11* | Exon9 | c.5292C>A | p.F1764L | Het | Missense | U | AD |
| 39 | *AIFM1* | Exon16 | **c.1783G>A** | **p.G595S** | hemi | Missense | U | XLR |
| 747 | *ADGRV1* | Exon45 ；Exon74 | **c.9701C>T ；c.15701A>G** | **p.Ala3234Val；p.Lys5234Arg** | Het；Het | Missense；Missense | U；U | AR |
| 325 | *BCOR* | Exon9 | **c.3925G>A** | **p.A1309T** | Hom | Missense | U | XLD |
| 608 | *CLDN14* | Exon3 | c.83C>T | p.Pro28Leu | Hom | Missense | U | AR |
| 548 | *CLRN1* | Exon1 | **c.121_136dup16** | **p.Val47fs** | Hom | Frameshift | P | AR |
| 650 | *CEACAM16* | Exon4 | **c.539C>A** | **p.Ser180Tyr** | Het | Missense | U | AD |
| 660 | *CABP2* | Exon5 | **c.487delG** | **p.Glu163Serfs** | Hom | Frameshift | LP | AR |
| 669 | *CD164* | Exon2 | **c.218T>A** | **p.Val73Asp** | Het | Missense | U | AD |
| 815 | *CCDC50* | Exon9 | **c.1156A>G** | **p.Met386Val** | Het | Missense | U | AD |
| 616 | *COL1A1* | Exon21 | **c.1442C>A** | **p.Pro481His** | Het | Missense | U | AD |
| 617 | *COL9A1* | Exon5；Exon5 | **c.347C>T；c.352C>T** | **p.Thr116Met；p.Arg118*** | Het；Het | Missense；Nonsense | U；P | AR |
| 626 | *COL2A1* | Exon51 | **c.3872C>T** | **p.Pro1291Leu** | Het | Missense | U | AD |
| 800 | *COL4A5* | Exon33 | **c.2858G>T** | **p.Gly953Val** | hemi | Missense | U | XLD |
| 541 | *DSPP* | Exon5 | **c.3660_3668del** | **p.Ser1229_Ser1231del** | Het | Indel-Inframe | U | AD |
| 26 | *EDNRB* | Exon1；Exon1 | **c.128C>G；c.25G>A** | **p.S43C；p.A9T** | Het；Het | Missense；Missense | U；U | AR |
| 387 | *ESRRB* | Exon9；Exon7 | **c.1114G>A；c.670C>G** | **p.E372K；p.L224V** | Het；Het | Missense；Missense | U；U | AR |
| 230 | *ERCC6* | Exon6；Exon6 | c.3166G>A；c.2387G>A | p.V1056I；p.G796D | Het；Het | Missense；Missense | LP；U | AR |
| 628 | *HOMER2* | Exon6 | **c.506T>C** | **p.Val169Ala** | Het | Missense | U | AD |
| 477 | *FLNA* | Exon14 | c.2122C>T | p.R708W | Hom | Missense | U | XLD |
| 601 | *FOXC1* | Exon1 | **c.745G>C** | **p.Gly249Arg** | Het | Missense | U | AD |
| 621 | *FLNB* | Exon23 | **c.9747_9748del/c.3964G>A** | **p.Val1322Ile** | Het | Missense | U | AD |
| 427 | *GRXCR1* | Exon1；Exon1 | **c.382C>T；c.79C>T** | p.Q128X；p.R27X | Het；Het | Nonsense；Nonsense | P；P | AR |
| 112 | *GAB1* | Exon9 | **c.1790_1791GT** | **(-)** | Hom | Indel-Inframe | U | AR |
| 674 | *GSDME* | Exon10 | c.1277_1279del | p.D426del | Het | Indel-Inframe | U | AD |
| 797 | *KCNQ4* | Exon14 | **c.2014G>A** | **p.Val672Met** | Het | Missense | U | AD |
| 635 | *LARS2* | Exon17；Exon12 | c.1987C>T；c.1196A>G | p.Arg663Trp；p.Glu399Gly | Het；Het | Missense；Missense | U；U | AR |
| 279 | *LRP2* | Exon45；Exon14 | **c.8501T>G；c.1897A>T** | **p.I2834S；p.T633S** | Het；Het | Missense；Missense | U；U | AR |
| 522 | *MARVELD2* | Exon2 | **c.782G>A** | **p.Gly261Glu** | Hom | Missense | U | AR |
| 696 | *MYH14* | Exon27 | **c.3698G>A** | **p.Arg1233Gln** | Het | Missense | U | AD |
| 742 | *NLRP3* | Exon1 | **c.226G>A** | **p.Ala76Thr** | Het | Missense | U | AD |
| 178 | *PRPS1* | Exon2 | c.217A>G | p.M73V | hemi | Missense | U | XLR |
| 46 | *PDE1C* | Exon13 | **c.1415T>C** | **p.F472S** | Het | Missense | U | AD |
| 481 | *PDZD7* | Exon15；Exon8 | **c.2602A>G；c.1267G>A** | **p.M868V；p.A423T** | Het；Het | Missense；Missense | U；U | AR |
| 537 | *P2RX2* | Exon11 | **c.1078G>A** | **p.Asp360Asn** | Het | Missense | U | AD |
| 732 | *POLG* | Intron2；Exon2 | **c.3482+8G>C； c.150_158del** | (-) ；p.Q53_Q55del | Het；Het | Intronic；Indel-Inframe | U；U | AR |
| 780 | *REST* | Exon4 | **c.2644T>A** | **p.Leu882Ile** | Het | Missense | U | AD |
| 551 | *SH3TC2* | Exon11；Exon11 | c.1378C>T；c.1211C>T | p.Gln460*；p.Pro404Leu | Het；Het | Nonsense；Missense | P；U | AR |
| 649 | *SIX5* | Exon2 | **c.1105C>A** | **p.Pro369Thr** | Het | Missense | U | AD |
| 482 | *TWIST1* | Exon1 | c.70C>G | p.P24A | Het | Missense | U | AD |
| 463 | *TMIE* | Exon2 | **c.201delG** | **p.L68Cfs*74** | Hom | Frameshift | L | AR |
| 459 | *TPRN* | Exon3；Exon2 | **c.2073+8G>T；c.1844_1845insGGAGGAGGA** | **splicing；p.E615delinsEEEE** | Het；Het | Splicing；Indel-Inframe | U；U | AR |
| 304 | *TNFRSF11A* | Exon5 | c.497C>T | p.T166M | Het | Missense | U | AD |
| 620 | *TRPV4* | Exon9 | **c.1529G>A** | **p.Arg510Gln** | Het | Missense | U | AD |
| 651 | *TBL1Y* | Intron8 | **c.457+1G>C** | **p.?** | hemi | Splicing | LP | XLR |
| 699 | *TCOF1* | Exon18 | **c.2933C>G** | **p.Ala978Gly** | Het | Missense | U | AD |
| 653 | *YAP1* | Exon1 | **c.233C>T** | **p.Ala78Val** | Het | Missense | U | AD |

*Abbreviations*: P, pathogenic; LP, likely pathogenic ; U: uncertain; AR, autosomal recessive; AD, autosomal dominant; XLR, X-linked recessive; XLD, X-dominant; Mi, mitochondria; CNV, copy number variation, Het, heterozygote; Hom, homozygote
